# Phase 1 study of pembrolizumab (MK-3475; anti-PD-1 monoclonal antibody) in Japanese patients with advanced solid tumors

**DOI:** 10.1007/s10637-016-0347-6

**Published:** 2016-03-22

**Authors:** Toshio Shimizu, Takashi Seto, Fumihiko Hirai, Mitsuhiro Takenoyama, Kaname Nosaki, Junji Tsurutani, Hiroyasu Kaneda, Tsutomu Iwasa, Hisato Kawakami, Kazuo Noguchi, Takashi Shimamoto, Kazuhiko Nakagawa

**Affiliations:** Department of Medical Oncology, Kindai University Faculty of Medicine, 377-2 Ohno-higashi, Osaka-sayama, Osaka 589-8511 Japan; Department of Thoracic Oncology, National Kyushu Cancer Center, 3-1-1 Notame, Minami-ku, Fukuoka, 811-1395 Japan; MSD K.K., Kitanomaru square, 1-13-12 Kudan-kita, Chiyoda-ku, Tokyo, 102-8667 Japan

**Keywords:** Pembrolizumab, Anti-PD-1 therapy, Pharmacokinetics, Phase I study, PD-L1

## Abstract

*Background* This phase I study evaluated the safety and tolerability, pharmacokinetics and pharmacodynamics, immunogenicity, and antitumor activity of pembrolizumab in Japanese patients with advanced solid tumors. *Methods* Following an initial dose and a 28-day rest (cycle 1), pembrolizumab was administered as an intravenous infusion at escalating doses (2 or 10 mg/kg) every 2 weeks (Q2W) until disease progression or unacceptable toxicity. Adverse events (AEs) were assessed using CTCAE v4.0, and tumor response was assessed using both RECIST v1.1 and immune-related response criteria (irRC). Full pharmacokinetic sampling was performed during cycle 1. *Results* Three patients received pembrolizumab at 2.0 mg/kg and seven at 10 mg/kg. No dose-limiting toxicities were observed during cycle 1. Eighty percent of patients experienced drug-related AEs (mostly grade 1 or 2); the most common drug-related AEs were nausea, malaise, pyrexia, and aspartate aminotransferase/alanine transaminase (AST/ALT) elevations (*n* = 2 each). No drug-related grade 4 or 5 AEs occurred. Immune-related AEs comprised grade 3 ALT elevation (*n* = 1), grade 3 AST elevation (*n* = 1), grade 1 pneumonitis (*n* = 1), and grade 1 thyroid-stimulating hormone elevation (*n* = 1). The safety and pharmacokinetic profiles of Japanese patients were similar to those previously reported for Caucasian patients. A partial tumor response was observed in one patient with non-small-cell lung cancer (NSCLC) and in one patient with melanoma. *Conclusions* Pembrolizumab at both 2 and 10 mg/kg Q2W was well tolerated in Japanese patients with advanced solid tumors and showed encouraging anti-tumor activity against melanoma and NSCLC.

## Introduction

The PD-1 pathway represents a major immune control switch that may be engaged by tumor cells to overcome active T-cell immune surveillance [1]. The ligands for PD-1 (PD-L1 and PD-L2) are constitutively expressed or can be induced in various tumors, including melanoma [2–5]. The high expression of PD-L1 (and to a lesser extent, PD-L2) on tumor cells is correlated with poor prognosis and poor survival in various cancer types, including renal cell carcinoma, pancreatic carcinoma, hepatocellular carcinoma, ovarian carcinoma, and non-small-cell lung cancer (NSCLC) [6–10]. Furthermore, it has been suggested that PD-1 regulates tumor-specific T-cell expansion in patients with melanoma [11]. Preclinical in vitro and in vivo experiments have shown that PD-1 and/or PD-L1 blockade using monoclonal antibodies enhances tumor cell-specific T-cell activation, cytokine production, anti-tumor effector mechanisms, and the clearance of tumor cells by the immune system [12–16]. PD-1 and PD-L1 inhibitors have validated PD-1 as an attractive target for clinical therapeutic intervention. PD-1 inhibition was tested in a clinical study of patients with a range of solid tumor types, and promising clinical activity was noted in multiple tumor types, including melanoma and NSCLC [17].

Pembrolizumab (formerly known as MK-3475) is a potent, highly selective, IgG4-k humanized monoclonal antibody that prevents PD-1 from binding with PD-L1 and PD-L2. This agent was generated by grafting the variable region sequences of a mouse antihuman PD-1 antibody onto a human IgG4-k isotype framework containing a stabilizing S228P mutation of the Fc region. Pembrolizumab exhibited high affinity for the PD-1 receptor, strong inhibition of PD-L1 and PD-L2, and robust activity in a functional ex vivo T-cell modulation assay using human donor blood cells (data on file; Merck & Co., Inc.).

A first-in-human phase 1 study was conducted to evaluate the safety, pharmacokinetics, and pharmacodynamics of pembrolizumab in non-Japanese patients with advanced solid tumors. No dose-limiting toxicities (DLTs) were observed, and the maximum administered dose (MAD) was 10 mg/kg every 2 weeks (Q2W). Pharmacokinetic and pharmacodynamic analyses showed that the lowest dose with the full potential for antitumor activity was 2 mg/kg every 3 weeks (Q3W) [18].

In the present study, the safety and tolerability, pharmacokinetics (PK), and immunogenicity of pembrolizumab were investigated in Japanese patients with advanced solid tumors. The tumor response to pembrolizumab was also evaluated as an exploratory objective.

## Materials and methods

### Patient eligibility

This study was conducted based on the Declaration of Helsinki and the Guidelines for the Clinical Evaluation Methods of Anti-Cancer Drugs in Japan (Japanese Ministry of Health, Labour, and Welfare notification, November 1, 2005). The study was approved by the institutional review board of each study site.

The main eligibility criteria were as follows: a histologically or cytologically confirmed diagnosis of locally advanced or metastatic solid tumors in a patient who had experienced disease progression while on standard therapy or in a patient intolerant of, or not eligible for standard therapy; a patient age of 20 years or older; an Eastern Cooperative Oncology Group (ECOG) performance status of 0 or 1; and adequate hematologic, hepatic, and renal functions. The exclusion criteria included the administration of chemotherapy, radiotherapy, or biological therapy in the 4 weeks (2 weeks for palliative radiotherapy and kinase inhibitors) prior to enrollment; previous treatment with a PD-1, PD-L1, or cytotoxic T-lymphocyte-associated protein 4 inhibitor; untreated and/or unstable central nervous system metastasis; or the presence of autoimmune disease.

All patients provided informed consent, and the study was conducted in accordance with current Good Clinical Practice standards. This study was registered at ClinicalTrials.gov as NCT01840579.

### Study design and evaluation

This study was an open-label, non-randomized, phase 1 study of pembrolizumab in Japanese patients with advanced solid tumors that was conducted at two sites in Japan. This study was designed to investigate the safety and tolerability, PK, immunogenicity, and anti-tumor activity of pembrolizumab monotherapy. Pembrolizumab was administered as a 30-min intravenous (i.v.) infusion at a dose of 2 or 10 mg/kg. Dose escalation was conducted using the conventional “3 + 3 design,” with cohorts of three patients sequentially enrolled at pembrolizumab doses of 2 and 10 mg/kg administered on days 1 and 28 and Q2W thereafter until disease progression or intolerable toxicity occurred. The initial 28 days after the first administration (cycle 1) were regarded as the DLT evaluation period. Three or six patients were enrolled at each dose based on the toxicity probability intervals [19]. In the DLT assessments, if none of the three patients or none/one of the six patients had a DLT at a certain dose, that dose was considered to be tolerable.

Adverse events (AEs) were graded using the National Cancer Institute Common Terminology Criteria for AEs, version 4.0. A DLT was defined as any of the following events occurring during cycle 1 (the initial 28 days): grade 4 neutropenia lasting for ≥7 days; grade 3 or 4 neutropenia with a fever >38.5 °C and/or infection requiring antibiotic or anti-fungal treatment; grade 4 thrombocytopenia; grade 4 non-hematologic toxicity; or a grade 3 non-hematologic toxicity (laboratory value) persisting for >1 week or requiring medical intervention.

Anti-tumor activity was evaluated at baseline and every 6 weeks according to the Response Evaluation Criteria In Solid Tumors, version 1.1 (RECIST v1.1) and the immune-related response criteria (irRC) [20].

### Pharmacokinetics and immunogenicity (presence of anti-drug antibody)

Blood samples for the PK analyses were collected predose, postdose (<30 min after infusion), and 6, 24, and 48 h after the start of the first infusion; on days 8, 15, 22, and 29 of cycle 1; predose and postdose in cycle 2 and every other cycle thereafter for the first 12 months; and 30 days after the last pembrolizumab dose. The pembrolizumab serum concentrations were quantified using a validated electrochemiluminescent assay (lower limit of quantification, 10 ng/mL). The PK data are described using a noncompartmental approach. Blood samples for anti-drug antibody (ADA) analyses were collected predose and 24 h after the start of the first infusion, predose in cycle 2 and every other cycle thereafter for the first 12 months, and 30 days after the last pembrolizumab dose.

The presence of ADA was determined using bridging electrochemiluminescence and was evaluated using the standard three-step method consisting of a screening test, a confirmation test, and an antibody titer test. ADA was determined to be positive if the result for at least one predose or postdose sample was positive on the confirmation test.

### PD-L1 expression

PD-L1 expression was measured using immunohistochemistry performed on formalin-fixed, paraffin-embedded tissue sections at QualTek Clinical Laboratories. PD-L1 positivity was defined as staining in ≥1 % of the tumor cells (modified proportion score) or stroma using immunohistochemistry with the PD-L1 22C3 antibody.

## Results

### Patient characteristics

Ten Japanese patients with advanced solid tumors were enrolled and were evaluated in this study; three patients were treated with 2 mg/kg Q2W and seven patients were treated with 10 mg/kg Q2W. The baseline characteristics of the patients are summarized in Table 1. The age range was 52.0–91.0 years (median: 62.0 years), and the most frequent solid tumors were NSCLC (5/10, 50.0 %) and melanoma (3/10, 30.0 %). The median number of prior chemotherapy regimens was 5 (range, 0–13). The median number of treatments was 4 (range, 1–18). At the time of data cutoff, nine of the ten patients had discontinued pembrolizumab treatment: one because of an adverse event (grade 4 cerebral infarction that was considered by the investigator to be unrelated to pembrolizumab treatment) and eight because of disease progression.

**Table 1:** Baseline patient characteristics

Characteristic	Value
Number of patients	10
Age, year	
Median	62.0
Range	52.0–91.0
Sex	
Male	5
Female	5
Weight, kg	
Median	61.7
Range	39.4–79.9
ECOG performance status	
0	4
1	6
Primary tumor	
NSCLC	5
Melanoma	3
Breast cancer	1
Extramammary Paget’s disease	1
Number of prior systemic therapies	
Median	5
Range	0–13

*ECOG* Eastern Cooperative Oncology Group

### Safety and tolerability

The DLTs occurring during cycle 1 were evaluated. One patient treated with pembrolizumab 10 mg/kg was excluded from the DLT evaluation because the patient discontinued the study because of disease progression during the DLT evaluation period. Therefore, that patient was replaced with a new patient for the DLT evaluation. No DLTs were observed in the three patients treated with 2 mg/kg or in the six patients treated with 10 mg/kg. The drug-related AEs reported for all treatment cycles at both dose levels are summarized in Table 2. Eighty percent of patients experienced drug-related AEs (mostly grade 1 or 2). The most common AEs related to pembrolizumab treatment were nausea, malaise, pyrexia, elevated alanine transaminase (ALT) levels, and elevated aspartate aminotransferase (AST) levels (2/10 [20 %] for each AE). No drug-related grade 4 or grade 5 AEs occurred in this study. None of the patients discontinued pembrolizumab treatment because of a drug-related AE. One patient (a 53-year-old man) with advanced NSCLC developed grade 3 ALT elevation, grade 3 AST elevation, and grade 1 pneumonitis (onset, day 42 for each); after the discontinuation of pembrolizumab treatment, he developed grade 3 hyponatremia (onset, day 56). The grade 3 ALT/AST elevations resolved after treatment with glycyrrhetinic acid, and the grade 3 hyponatremia resolved with sodium chloride supplement. Overall, one case each of grade 3 ALT elevation, grade 3 AST elevation, grade 1 pneumonitis, and grade 1 thyroid-stimulating hormone (TSH) elevation were reported as immune-related AEs by the investigators.

**Table 2:** All grades of adverse events related to pembrolizumab treatment

	2 mg/kg, Q2W (*n* = 3)		10 mg/kg, Q2W (*n* = 7)		Total (*n* = 10)	
	All Grades	Grade 3	All Grades	Grade 3	All Grades	Grade 3
Blood and lymphatic system disorders						
Anemia	0	0	1	0	1	0
Neutropenia	0	0	1	0	1	0
Thrombocytopenia	0	0	1	0	1	0
Gastrointestinal disorders						
Nausea	1	1	1	0	2	0
General disorders and administration site conditions						
Malaise	1	0	1	0	2	0
Pyrexia	1	0	1	0	2	0
Fatigue	1	0	0	0	1	0
Infections and infestations						
Infection	0	0	1	0	1	0
Investigations						
ALT elevation	0	0	2	1	2	1
AST elevation	0	0	2	1	2	1
Blood cholesterol elevation	1	0	0	0	1	0
Blood TSH elevation	0	0	1	0	1	0
Weight reduction	0	0	1	0	1	0
Metabolism and nutrition disorders						
Dehydration	0	0	1	0	1	0
Hyperglycemia	0	0	1	0	1	0
Hyperkalemia	0	0	1	0	1	0
Hypertriglyceridemia	1	0	0	0	1	0
Hypokalemia	0	0	1	0	1	0
Hyponatremia	0	0	1	1	1	1
Respiratory, thoracic, and mediastinal disorders						
Exertional dyspnea	0	0	1	0	1	0
Pneumonitis	0	0	1	0	1	0
Skin and subcutaneous tissue disorders						
Drug eruption	0	0	1	0	1	0
Rash	0	0	1	0	1	0
Urticaria	0	0	1	0	1	0

AEs related to pembrolizumab were grade 1, 2, or 3 in severity, with no grade 4 or 5 AEs reported

### Pharmacokinetic and immunogenicity evaluation

**Fig. 1:**
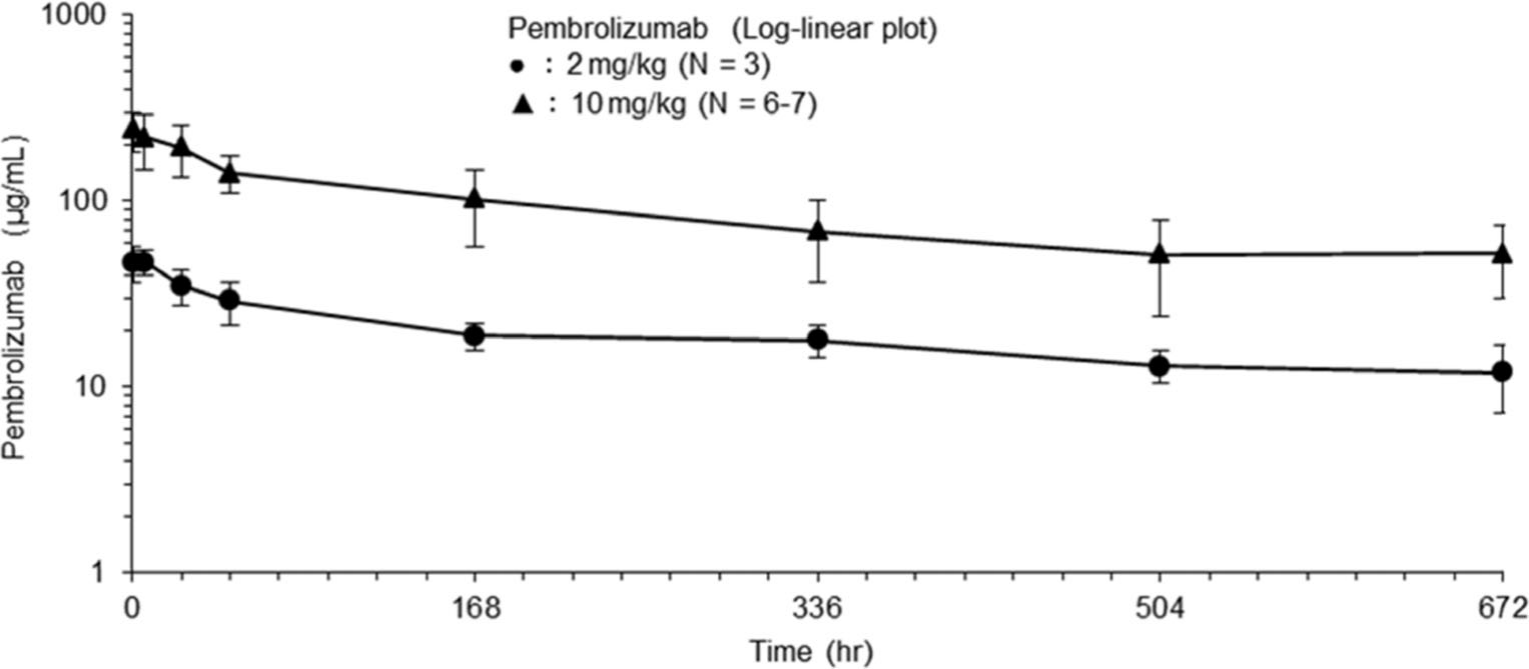
Serum concentration–time profiles of pembrolizumab following the first administration. Symbols and error bars indicate the mean ± SD

**Fig. 2:**
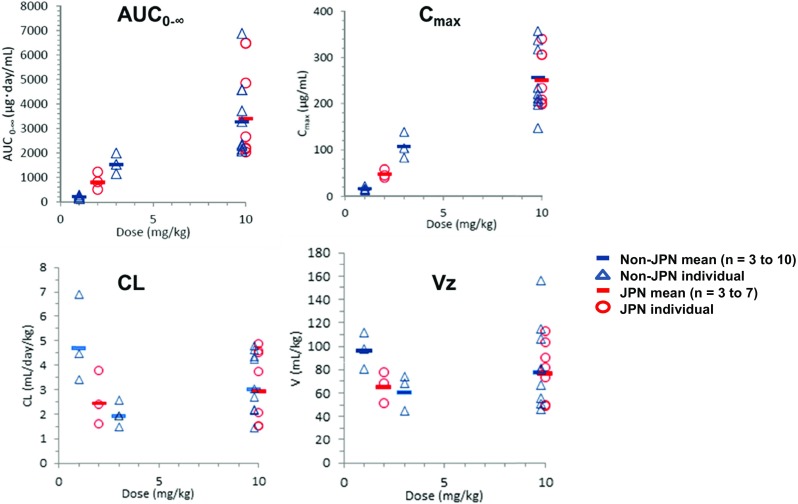
PK parameters versus dose following the first administration of pembrolizumab in Japanese and non-Japanese patients with advanced solid tumors. *AUC*_*0-∞*_ total area under the concentration–time curve, *C*_*max*_ maximum concentration, *CL* clearance, *Vz* the terminal phase volume

The mean serum concentration profiles for pembrolizumab are shown in Fig. 1, and descriptive statistics for the PK parameters are given in Table 3 and Fig. 2. The serum concentration of pembrolizumab following the first i.v. administration decreased slowly in Japanese patients with advanced solid tumors. The geometric mean terminal half-lives (t_1/2_) for pembrolizumab following the first i.v. administration were 18.4 and 18.1 days at doses of 2 and 10 mg/kg, respectively, in Japanese patients. For doses of 2 and 10 mg/kg, respectively, the geometric mean (and coefficient of variation) values for the clearance (CL) were 2.46 (45 %) and 2.93 (56 %) mL/day/kg and for the terminal phase volume (Vz) were 65.3 (21 %) and 76.5 (34 %) mL/kg. The t_1/2_, CL, and Vz values were very similar for doses of 2 and 10 mg/kg in Japanese patients with advanced solid tumors. Exposure to pembrolizumab, as reflected by the total area under the concentration–time curve (AUC_0-∞_) and maximum concentration (C_max_), following the first i.v. administration of pembrolizumab at doses of 2 and 10 mg/kg generally increased in a dose-proportional manner in Japanese patients. The PK profiles of pembrolizumab, which has a low clearance, a limited volume of distribution, and a long t_1/2_, are typical of therapeutic antibodies. The trough concentration increased during repeated dosing at 2 or 10 mg/kg Q2W. Based on the t_1/2_ (18 days) and the dose interval (2 weeks), approximately 3.5 months (cycle 8) were considered necessary to achieve a steady state. However, only two patients treated with 2 mg/kg and one patient treated with 10 mg/kg were still receiving pembrolizumab at cycle 8. Considering the limited PK data available for doses of 2 and 10 mg/kg beyond cycle 8, the steady state trough concentration could not be determined based on the observed PK data.

**Table 3:** Summary of pharmacokinetic parameters following the first dose (2 or 10 mg/kg) of pembrolizumab in Japanese patients with advanced solid tumors

Dose	C_max,_ μg/mL, GM (CV%)	T_max,_ days, median (range)	AUC_0–28_, μg day/mL, GM (CV%)	AUC_0-∞_, μg day/mL, GM (CV%)	t_1/2,_ days, GM (CV%)	Vz, mL/kg, GM (CV%)	CL, mL/day/kg, GM (CV%)
2 mg/kg (*n* = 3)	47.4 (19)	0.22 (0.002–0.23)	507 (20)	812 (45)	18.4 (56)	65.3 (21)	2.46 (45)
10 mg/kg (*n* = 7)	250 (23)	0.01 (0.001–0.23)	2219 (32)	3410 (56)	18.1 (68)	76.5 (34)	2.93 (56)

*C*_*max*_ maximum observed serum concentration, *T*_*max*_ time of maximum observed serum concentration, *AUC*_*0–28*_ area under the concentration–time curve from day 0 to day 28, *AUC*_*0–∞*_ area under the concentration–time curve from day 0 to infinity, *t*_*1/2*_ elimination half-life, *Vz* the terminal phase volume, *CL* clearance, *GM* geometric mean, *CV* coefficient of variation

ADA was not detected at any time in screening assays from predose through to cycle 18 (day 140), except in a predose assay performed during cycle 1 in one patient; however, the results of the confirmatory assay were negative for this patient.

### Antitumor activity and PD-L1 expression

**Fig. 3:**
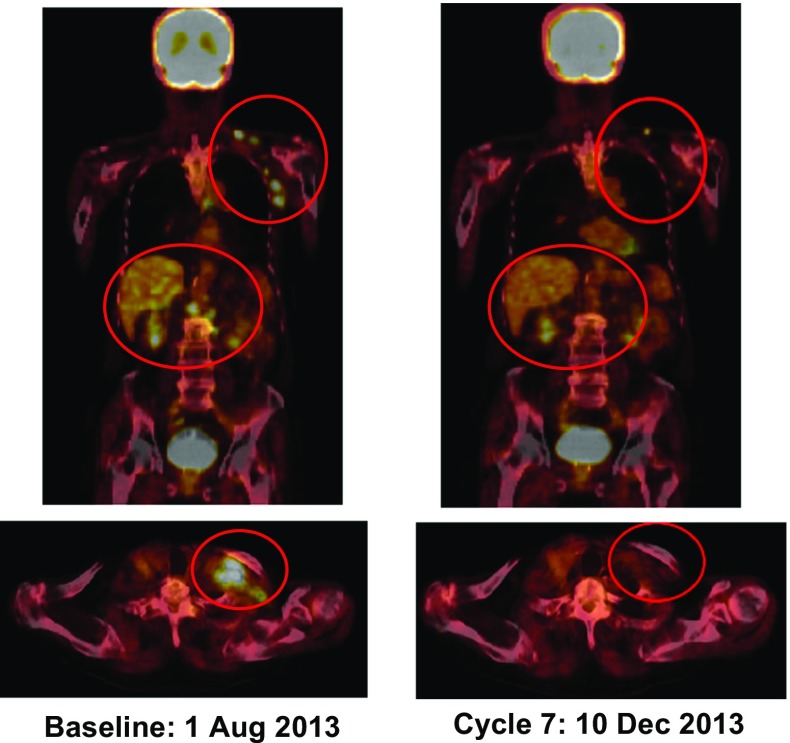
PET-CT images taken before the start of treatment and after cycle 7 show evidence of antitumor activity. A rapid and durable partial response according to irRC was observed in a 91-year-old man with advanced metastatic acral lentiginous melanoma who had active disease in the liver and multiple lymph nodes when treatment with pembrolizumab at 10 mg/kg Q2W was initiated

As an exploratory objective, the tumor response to pembrolizumab was evaluated according to the RECIST v1.1 and irRC criteria. One patient with extramammary Paget’s disease was excluded from the tumor response assessment because the patient did not have any measurable lesions at the study baseline. Among the nine patients who were evaluated, partial responses (as determined by an investigator review according to RECIST v1.1) were observed in two patients (22.2 %) treated with pembrolizumab 10 mg/kg Q2W; one patient (a 91-year-old man) had metastatic melanoma (time to response: 46 days), and the other (a 53-year-old man) had NSCLC (time to response: 41 days). The two patients also had partial responses as determined by an investigator review according to irRC. The patient with advanced NSCLC developed immune-related AEs (grade 3 ALT/AST elevations and grade 1 pneumonitis) at the same time as the observation of the partial response. At the time of data cutoff, the patient with advanced metastatic melanoma who had achieved a partial response was continuing to receive pembrolizumab treatment because, despite disease progression according to RECIST v1.1, disease progression was not evident according to irRC (response duration: more than 225 days) (Fig. 3). Tumor tissue from five patients was available to undergo a PD-L1 expression immunohistochemistry assay. PD-L1 expression was positive in tumor samples from two patients and negative in tumor samples from three patients. No responses were observed in the two patients with PD-L1-positive tumors by an investigator review whereas a partial response was observed in one of the three patients with PD-L1-negative tumors.

## Discussion

The primary objective of the present study was to investigate the safety and tolerability of single-agent pembrolizumab administered in Japanese patients with advanced solid tumors. The dosing schedules in the present study were selected based on the results of a phase 1 study of pembrolizumab in non-Japanese patients with advanced solid tumors. The previous study showed that the MAD was 10 mg/kg Q2W, and the lowest dose with the full potential for antitumor activity was 2 mg/kg Q3W [18]. No DLTs were observed at either dose in the present study. The most common drug-related AEs were nausea, malaise, pyrexia, and AST/ALT elevations (*n* = 2 each). One case each of grade 3 ALT elevation, grade 3 AST elevation, grade 1 pneumonitis, and grade 1 TSH elevation were reported as immune-related AEs. The AEs observed in the present study have also been reported for clinical studies of pembrolizumab in non-Japanese patients. Although the number of Japanese patients was limited, the safety profile of pembrolizumab in Japanese patients with advanced solid tumors in the present study was generally consistent with that observed previously in non-Japanese patients. Drug-induced autoimmune-like toxicities have been observed in patients treated with immune checkpoint inhibitors (such as those used in anti-PD-1 therapy) through the infiltration of immune cells into normal noncancerous tissues. Immune-related AEs can affect multiple organs such as the skin, bowel, kidney, peripheral and central nervous system, liver, lymph nodes, eyes, pancreas, and endocrine tissues. Steroid therapy is recommended for the management of immune-related AEs [21, 22]. In the present study, most AEs were of grade 1 or 2 and were manageable by interrupting pembrolizumab treatment and/or medical intervention without steroid therapy.

Pembrolizumab exposure at a dose of 10 mg/kg was generally similar for Japanese and non-Japanese patients with advanced solid tumors (AUC_0-∞_: 3410 vs. 3270 μg day/mL, C_max_: 250 vs. 256 μg/mL, respectively). The mean CL, t_1/2_, and Vz values were generally similar between the two populations for doses in the range 1–10 mg/kg. In addition, the individual values for AUC_0-∞_, C_max_, t_1/2_, Vz, and CL for doses in the range 1–10 mg/kg overlapped (Fig. 2) [18]. These results indicated that the PK of pembrolizumab in Japanese patients with advanced solid tumors was generally similar to that in non-Japanese patients with advanced solid tumors.

As an exploratory analysis, the tumor responses were also evaluated. Partial responses were observed in two of the nine patients (22.2 %) treated with 10 mg/kg Q2W by an investigator review according to both RECIST v1.1 and irRC criteria. One of the five patients with advanced NSCLC and one of the three patients with advanced melanoma achieved partial responses. A durable response (response duration of more than 225 days according to irRC) was observed in one patient with metastatic acral lentiginous melanoma, which is the most common subtype of melanoma in Asian countries (superficial spreading melanoma is more common in Caucasians). This result is encouraging for the further development of pembrolizumab treatment for melanoma in Japan.

Because of the limited number of patients and tumor samples, a relationship between tumor response and PD-L1 expression was not observed in the present study. Recent data have shown that PD-L1 expression, microsatellite instability high, increased mutation burden, and the immune gene expression signature are correlated with the improved efficacy of pembrolizumab [23–26], indicating the importance of biomarker development to identify patients suitable for anti-PD-1 and anti-PD-L1 therapy. Based on the safety data from this phase 1 study, Japanese patients are currently being enrolled in late-phase global studies of pembrolizumab for the treatment of various types of tumors, such as NSCLC, gastric cancer, head and neck cancer, bladder cancer, and colorectal cancer. Further biomarker analyses in these clinical studies are on-going.

Recent randomized studies of pembrolizumab demonstrated that no clinically meaningful differences in the safety and efficacy of pembrolizumab were evident between 2 mg/kg Q3W and 10 mg/kg Q3W or between 10 mg/kg Q3W and 10 mg/kg Q2W [27–31]. The administration of pembrolizumab at 2 mg/kg Q3W is currently approved in the United States and other countries for the treatment of patients with unresectable or metastatic melanoma or metastatic NSCLC expressing PD-L1.

In conclusion, pembrolizumab at dosages of 2 and 10 mg/kg Q2W was well tolerated in Japanese patients with advanced solid tumors and showed encouraging anti-tumor activity against melanoma and NSCLC.

## References

[1] Disis ML (2010). Immune regulation of cancer. J Clin Oncol.

[2] Dong H, Strome SE, Salomao DR, Tamura H, Hirano F, Flies DB (2002). Tumor-associated B7-H1 promotes T-cell apoptosis: a potential mechanism of immune evasion. Nat Med.

[3] Sharpe AH, Freeman GJ (2002). The B7-CD28 superfamily. Nat Rev Immunol.

[4] Brown JA, Dorfman DM, Ma F-R, Sullivan EL, Munoz O, Wood CR (2003). Blockade of programmed death-1 ligands on dendritic cells enhances T cell activation and cytokine production. J Immunol.

[5] Francisco LM, Sage PT, Sharpe AH (2010). The PD-1 pathway in tolerance and autoimmunity. Immunol Rev.

[6] Thompson RH, Dong H, Lohse CM, Leibovich BC, Blute ML, Cheville JC (2007). PD-1 is expressed by tumor-infiltrating immune cells and is associated with poor outcome for patients with renal cell carcinoma. Clin Cancer Res.

[7] Nomi T, Sho M, Akahori T, Hamada K, Kubo A, Kanehiro H (2007). Clinical significance and therapeutic potential of the programmed death-1 ligand/programmed death-1 pathway in human pancreatic cancer. Clin Cancer Res.

[8] Gao Q, Wang X-Y, Qiu S-J, Yamato I, Sho M, Nakajima Y (2009). Overexpression of PD-L1 significantly associates with tumor aggressiveness and postoperative recurrence in human hepatocellular carcinoma. Clin Cancer Res.

[9] Hamanishi J, Mandai M, Iwasaki M, Okazaki T, Tanaka Y, Yamaguchi K (2007). Programmed cell death 1 ligand 1 and tumor-infiltrating CD8+ T lymphocytes are prognostic factors of human ovarian cancer. Proc Natl Acad Sci U S A.

[10] Mu CY, Huang JA, Chen Y, Chen C, Zhang XG (2011). High expression of PD-L1 in lung cancer may contribute to poor prognosis and tumor cells immune escape through suppressing tumor infiltrating dendritic cells maturation. Med Oncol.

[11] Fourcade J, Kudela P, Sun Z, Shen H, Land SR, Lenzner D (2009). PD-1 is a regulator of NY-ESO-1-specific CD8+ T cell expansion in melanoma patients. J Immunol.

[12] Gao Q, Wang XY, Qiu SJ, Yamato I, Sho M, Nakajima Y (2009). Overexpression of PD-L1 significantly associates with tumor aggressiveness and postoperative recurrence in human hepatocellular carcinoma. Clin Cancer Res.

[13] Blank C, Mackensen A (2007). Contribution of the PD-L1/PD-1 pathway to T-cell exhaustion: an update on implications for chronic infections and tumor evasion. Cancer Immunol Immunother.

[14] Tsushima F, Tanaka K, Otsuki N, Youngnak P, Iwai H, Omura K (2006). Predominant expression of B7-H1 and its immunoregulatory roles in oral squamous cell carcinoma. Oral Oncol.

[15] Iwai Y, Ishida M, Tanaka Y, Okazaki T, Honjo T, Minato N (2002). Involvement of PD-L1 on tumor cells in the escape from host immune system and tumor immunotherapy by PD-L1 blockade. Proc Natl Acad Sci U S A.

[16] Sznol M, Powderly JD, Smith DC, Brahmer JR, Drake CG, McDermott DF (2010). Safety and antitumor activity of biweekly MDX-1106 (anti-PD-1, BMS-936558/ONO-4538) in patients with advanced refractory malignancies [Abstract]. J Clin Oncol.

[17] Brahmer JR, Drake CG, Wollner I, Powderly JD, Picus J, Sharfman WH (2010). Phase I study of single-agent anti-programmed death-1 (MDX-1106) in refractory solid tumors: safety, clinical activity, pharmacodynamics, and immunologic correlates. J Clin Oncol.

[18] Patnaik A, Kang SP, Rasco D, Papadopoulos KP, Elassaiss-Schaap J, Beeram M (2015). Phase I study of pembrolizumab (MK-3475; anti-PD-1 monoclonal antibody) in patients with advanced solid tumors. Clin Cancer Res.

[19] Ji Y, Li Y, Nebiyou Bekele B (2007). Dose-finding in phase I clinical trials based on toxicity probability intervals. Clin Trials.

[20] Wolchok JD, Hoos A, O’Day S, Weber JS, Hamid O, Lebbé C (2009). Guidelines for the evaluation of immune therapy activity in solid tumors: immune-related response criteria. Clin Cancer Res.

[21] Chen TW, Razak AR, Bedard PL, Siu LL, Hansen AR (2015). A systematic review of immune-related adverse event reporting in clinical trials of immune checkpoint inhibitors. Ann Oncol.

[22] Weber JS, Kähler KC, Hauschild A (2012). Management of immune-related adverse events and kinetics of response with ipilimumab. J Clin Oncol.

[23] Garon EB, Rizvi NA, Hui R, Leighl N, Balmanoukian AS, Eder JP (2015). Pembrolizumab for the treatment of non-small-cell lung cancer. N Engl J Med.

[24] Le DT, Uram JN, Wang H, Bartlett BR, Kemberling H, Eyring AD (2015). PD-1 Blockade in tumors with mismatch-repair deficiency. N Engl J Med.

[25] Rizvi NA, Hellmann MD, Snyder A, Kvistborg P, Makarov V, Havel JJ (2015). Cancer immunology. Mutational landscape determines sensitivity to PD-1 blockade in non-small cell lung cancer. Science.

[26] Ribas A, Robert C, Hodi FS, Wolchok JD, Joshua AM, Hwu WJ et al. (2015) Association of response to programmed death receptor 1 (PD-1) blockade with pembrolizumab (MK-3475) with an interferon-inflammatory immune gene signature [Meeting Abstract]. J Clin Oncol 33 (suppl; abstr 3001)

[27] Ribas A, Puzanov I, Dummer R, Schadendorf D, Hamid O, Robert C (2015). Pembrolizumab versus investigator-choice chemotherapy for ipilimumab-refractory melanoma (KEYNOTE-002): a randomised, controlled, phase 2 trial. Lancet Oncol.

[28] Robert C, Joshua AM, Weber JS, Ribas A, Hodi FS, Kefford RF et al. (2014) Pembrolizumab (Pembro; MK-3475) for advanced melanoma (MEL): randomized comparison of two dosing schedules. Ann Oncol 25 (suppl 4): doi: 10.1093/annonc/mdu438.42

[29] Robert C, Schachter J, Long GV, Arance A, Grob JJ, Mortier L (2015). Pembrolizumab versus ipilimumab in advanced melanoma. N Engl J Med.

[30] Robert C, Ribas A, Wolchok JD, Hodi FS, Hamid O, Kefford R (2014). Anti-programmed-death-receptor-1 treatment with pembrolizumab in ipilimumab-refractory advanced melanoma: a randomised dose-comparison cohort of a phase 1 trial. Lancet.

[31] Herbst RS, Baas P, Kim DW, Felip E, Pérez-Gracia JL, Han JY (2015). Pembrolizumab versus docetaxel for previously treated, PD-L1-positive, advanced non-small-cell lung cancer (KEYNOTE-010): a randomised controlled trial. Lancet.

